# Development of a vasopressor control module for testing hemorrhagic shock resuscitation controllers

**DOI:** 10.1186/s12938-026-01552-3

**Published:** 2026-03-26

**Authors:** David Berard, Michael D. Lopez, Austin Ruiz, Jonathan Marrero Bermudez, Rachel Gathright, Tina M. Rodgers, Sofia I. Hernandez Torres, Caroline Gusson Shimoura, Evan Ross, Eric J. Snider

**Affiliations:** 1 Organ Support & Automation Technologies Group, U.S. Army Institute of Surgical Research, JBSA Fort Sam Houston, Texas, 78234 USA; 2 Department of Surgery, Long School of Medicine, UT Health San Antonio, San Antonio, TX USA

**Keywords:** Closed-loop controllers, Vasopressor therapy, Hardware-in-loop, Automation, Hemorrhagic shock, Fluid resuscitation, Product testing

## Abstract

**Introduction:**

Patients in hemorrhagic shock who are unresponsive to fluid administration can potentially benefit from and may even require vasopressor therapy. However, manual vasopressor titration in mass casualty or resource-limited settings can be arduous, increasing the risk of under- or over-treatment. Closed-loop vasopressor adaptive resuscitation controllers (V-ARCs) offer a potential solution, but their development is hindered when the iterative tuning and design process is primarily dependent on large-scale animal studies. To solve this problem, we developed a Vasopressor Control Module (VCM) for a hardware-in-loop automated testbed for resuscitation controllers (HATRC) to enable the systematic evaluation of V-ARCs.

**Methods and Results:**

The VCM’s design was informed by vasopressor administration data captured in a hemorrhagic shock swine model, which revealed four key physiological variables that defined the hemodynamic response to vasopressor infusion: lag time, real response, overshoot, and pressure–time responsiveness. The incorporation of these variables enabled the VCM to replicate physiological variability, dose-dependent responsiveness, and disturbance conditions representative of the clinical setting. Proof-of-concept testing was achieved by comparing multiple V-ARC designs under different testing conditions and successfully differentiating their performance.

**Conclusions:**

This work establishes a flexible, physiologically grounded platform for vasopressor controller development, reducing dependence on animal testing while enabling rapid and robust controller evaluation. Future work will expand physiological modeling, incorporate additional hemodynamic variables, and support multiagent resuscitation.

## Introduction

Successful treatment of hemorrhagic shock depends on rapid control of the source of the bleed, followed by fluid volume replenishment to ensure adequate organ perfusion [1, 2]. While the current standard of care relies on the administration of fluids, some patients are not volume responsive and exhibit a progressive physiological decline despite aggressive fluid infusion [3]. For those patients, early adjunctive administration of vasopressors [4–6] may provide hemodynamic support when blood products are not effective, while also mitigating the potential for fluid overload.

Vasopressors are powerful vasoactive drugs that require extremely close management by skilled medical providers. Under resource constrained conditions and/or during mass-casualty (MASCAL) events, close attention from skilled staff may not be available, meaning that patients who require vasopressor therapy may not have access to the lifesaving care they need. Even in well-resourced clinical settings, where patients can be closely monitored, imprecise titration can lead to excessive vasopressor exposure causing deleterious effects on renal and cardiac function.

To solve this problem, we propose the development of vasopressor adaptive resuscitation controllers (V-ARCs), physiological closed-loop controllers (PCLC) that balance the infusion of vasopressors and fluids to maintain a patient’s blood pressure. Libert et al. performed a pilot study of fluid resuscitation on swine subjects during hemorrhagic shock, evaluating closed-loop resuscitation performance using fluids alone, fluids with moderate norepinephrine infusion, and fluids with high norepinephrine infusion. Although time spent within the target pressure range showed no difference between treatments, swine resuscitated with norepinephrine required less fluid overall and had less hemodilution as a result. Closed-loop resuscitation also showed no difference in performance from manual resuscitation by a dedicated physician when continuous pressure readings were available [7]. Other groups have also worked on automating vasopressor delivery with promising results [8–11]. While preclinical animal research is the gold-standard for demonstrating the safety and efficacy of a therapeutic intervention prior to use in humans, animal testing is expensive, time consuming, and ethically complex. Non-animal models have been developed for studying automated vasopressor administration and include in silico models, such as digital twins, that use computer and mathematical analysis to simulate cardiovascular physiology as well as vasopressor responsiveness [8, 12–18]. Bighamian et al. developed a tool for predicting the dose–response of key cardiovascular parameters such as stroke volume, blood pressure and heart rate as a function of vasopressor infusion [19]. However, a common drawback among some in silico models is that they require extensive computing resources to run effectively, are highly dependent on both the quality and quantity of the training data, face generalizability challenges, and are extremely sensitive to initial conditions [20–22].

Other alternatives to preclinical animal testing are benchtop models for in vitro experiments that are inexpensive, allow rapid prototyping, and have no ethical issues to navigate. For this work, we used our existing hardware-in-loop automated testbed for resuscitation controllers (HATRC) [23], a bulk model for hemodynamic physiology that can simulate the mean arterial pressure (MAP) response to whole blood and/or crystalloid infusion [24, 25]. The platform offers both customizability and repeatability for comparing resuscitation controller designs, something currently unavailable with typical in silico models [26].

In this study, we develop an extension to HATRC that would allow it to simulate a patient’s response to vasoactive medications. This extension, called the Vasopressor Control Module (VCM), aims to recreate the physiological response to vasopressor infusion in a benchtop model, thus reducing the need for animal testing. Overall, our group proposes the following objectives to meet for this study:

- To develop hardware that simulates a patient’s MAP response to vasopressors based on post hoc animal data analyses.
- To demonstrate a functional VCM integrated with the group’s HATRC platform that can produce pressure responses that mimic key features observed in the animal data.
- To determine if the system can differentiate the performance of two similar V-ARC designs in identical scenarios.

## Results

The development and testing of the VCM module are presented first with the analysis of pressure data collected during vasopressor administration in a swine model of hemorrhagic shock, and a selection of key physiological response variables are introduced. Next, is a description of the VCM and its characterization along with a similar overview of a mechanical disturbance generator module (DGM). Lastly, proof-of-concept testing of two decision table-driven V-ARCs is presented and quantitatively analyzed using controller performance metrics to differentiate their performance.

### Animal data analysis

Development of the VCM started with an analysis of swine data with the goal of characterizing the hemodynamic changes that occurred from vasopressor administration during hemorrhagic shock (see methods). A pump-controlled hemorrhage was used to reach a MAP of 35 mmHg, followed by titration of a 4 mcg/mL NE solution until MAP was restored to at least 65 mmHg, then a single weaning step was attempted at the end to observe the hemodynamic effects of a reduction in vasopressor infusion rate. The dosage of drug was controlled by adjusting the infusion rate of the solution.

**Fig. 1:**
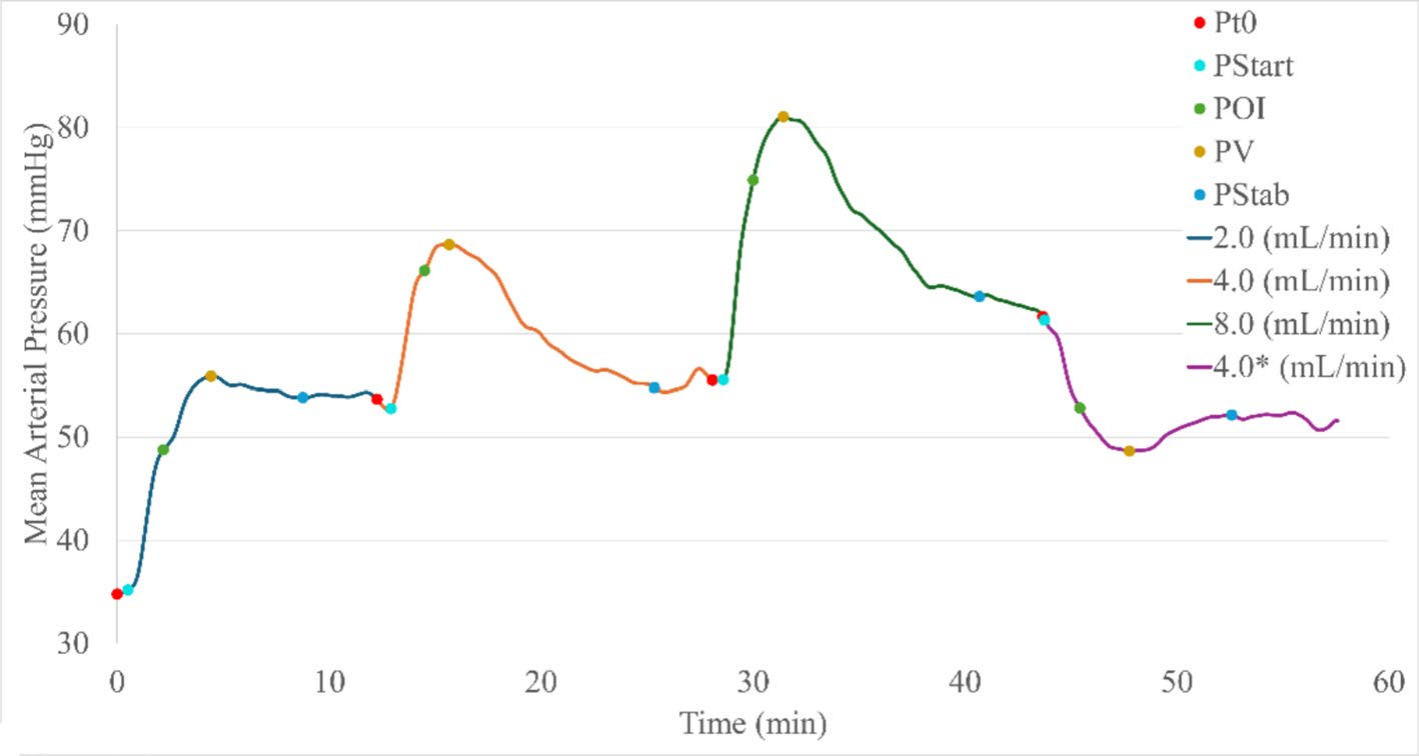
Representative mean arterial pressure (MAP) waveform variable analyses during vasopressor therapy. Combined plot of escalating NE vasopressor infusion rates with unique points of interest marked for each rate. (* indicates the rate was reached following an infusion rate reduction)

The transient MAP signal during vasopressor administration was analyzed to extract key fiducial waveform markers as well as four derived metrics to characterize the pressure and time responsiveness to the vasopressor therapy. Key markers included the pressure at the moment the infusion rate change occurs (P_t0_), the pressure when a noticeable response is detected (P_Start_), a “point of inflection” where the first derivative of the pressure signal drops sufficiently below an observed maximum (POI), the pressure response vertex (P_V_), and the relative stable pressure (P_Stab_) following each infusion rate. A representative subject waveform is shown in Fig. 1 with the points of interest marked; each marker is summarized in methods Table 2. From this initial broad array, we identified four essential physiological metrics that could be used to describe any given animal’s hemodynamic response to an increase in the rate of NE infusion: (i) lag time, (ii) real response, (iii) overshoot and (iv) pressure–time responsiveness (dMAP/dt). An illustrative diagram of the derived features can be seen in Fig. 10 in the methods as well as a detailed description of each metric in methods Table 3.

**Fig. 2:**
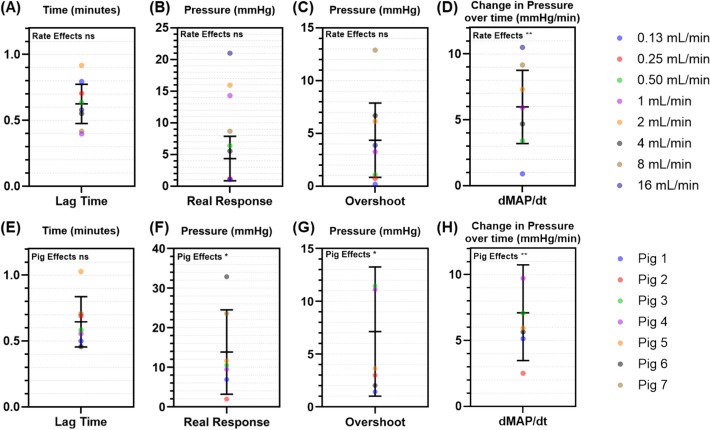
Evaluation of the effect of subject variability and vasopressor infusion rate on response variables. The effect of **A**–**D** vasopressor infusion rate (mL/min) over 8 steps and E–H subject variability across 7 pigs are shown for **A**,**E** Lag Time (mins), **B**,**F** Real Response (mmHg), **C**,**G** Overshoot (mmHg), and **D**,**H** Pressure–Time Responsiveness (dMAP/dt, mmHg/min). Responses for infusion rate or subject are shown with average and standard deviations for each. Statistical significance for the effect of either vasopressor infusion rate or subject variability is shown based on 2-way ANOVA analysis (ns = not significant; * = *p* < 0.05; ** = *p* < 0.01).

To aid in the design of the VCM, two critical questions to answer were (i) whether any of these extracted variables were a function of vasopressor concentration or (ii) whether any variable was significantly altered by inter-subject variability. Statistical analyses across the entire dataset were performed for each metric to answer these questions. Overall, we found that vasopressor infusion rate only significantly impacted pressure–time responsiveness, while the other three variables were not significantly impacted (Fig. 2A–D). Conversely, inter-subject variability had a larger effect on three of the variables, with only lag time not being significantly impacted (Fig. 2E–H). This analysis allowed us to tailor the disturbance and variability of the test platform response and specify the conditions affecting it (e.g., subject variability or real-time rate dependence).

**Fig. 3:**
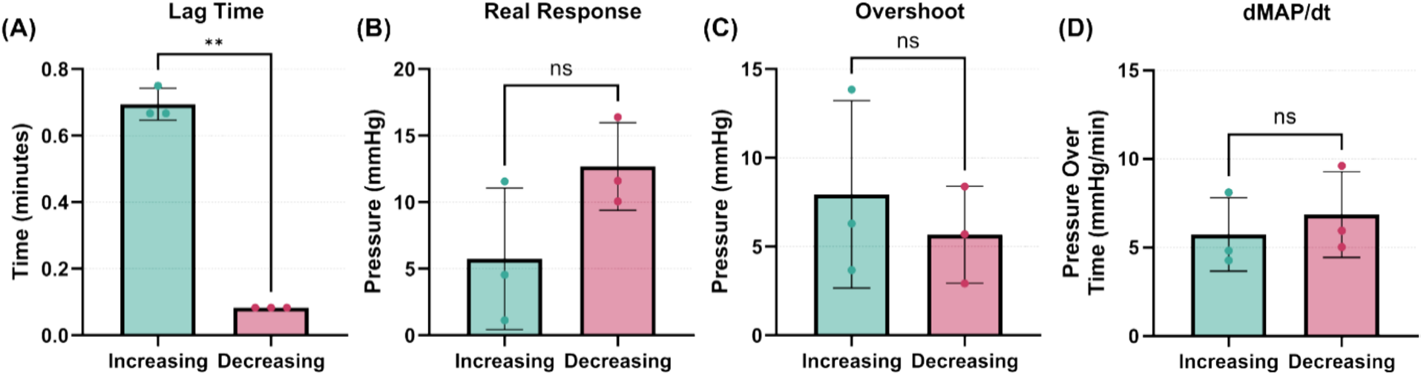
Different effects on response variables when increasing or decreasing vasopressor rates. Results are shown for **A** Lag Time (mins), **B** Real Response (mmHg), **C** Overshoot (mmHg), and **D** Pressure–Time Responsiveness (dMAP/dt, mmHg/min). Average values for matched vasopressor infusion rates are shown (*n* = 3 swine subjects) with error bars denoting standard deviation. Welch’s t-test was used to assess statistically significant differences between increasing and decreasing vasopressor infusion rate (ns = not significant; ** = *p* < 0.01)

In addition, we assessed the impact of increasing or decreasing vasopressor infusion rate on each of these response variables as the VCM can potentially be configured for testing vasopressor weaning off response (Fig. 3). Lag Time was the only variable with a significant difference; the other three metrics were not significant, indicating there was no dependence on whether the rate was increasing or decreasing and enabling symmetric bi-directional response profiles to be used. It should be noted, however, that decreasing rate steps were taken less frequently (only 6/7 subjects received a single rate reduction step and one dataset was excluded due to missing data), so only NE concentrations with balanced increasing and decreasing infusion rate directions were used in this analysis, excluding two additional subjects and resulting in an overall low sample size (*n* = 3). Thus, more data are needed to further refine this analysis.

### Vasopressor control module

**Fig. 4:**
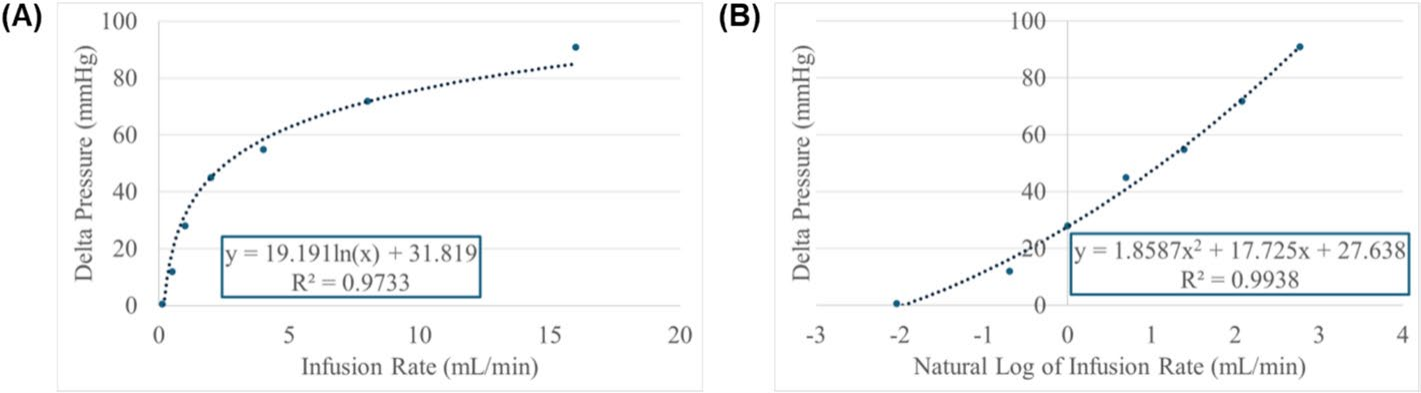
Average delta pressure response to vasopressor infusion rate characterization. **A** Original data with logarithmic regression overlayed. **B** Transformed data with quadratic regression overlayed

The VCM comprises a microcontroller, stepper motor, and needle valve which is plumbed into the fluid circulating portion of the HATRC loop. Flow is occluded as the valve is tightened, increasing the flow resistance and raising the pressure (see Methods subsection “Modifications of the Test Platform” for more information). The response variables were used when programming the microcontroller to achieve the desired response. To convert the administered infusion rate to a pressure delta, we first characterized the Real Response of the animal data using logarithmic regression as shown in Fig. 4A. To improve the accuracy of the characterization, we transformed the data by taking the natural log of the infusion rate followed by fitting the data with a quadratic regression shown in Fig. 4B. The resulting R^2^ value of the quadratic fit on the transformed data increased to above 0.99. This function was used to predict the net pressure change induced by any given infusion rate administered by the V-ARC.

**Fig. 5:**
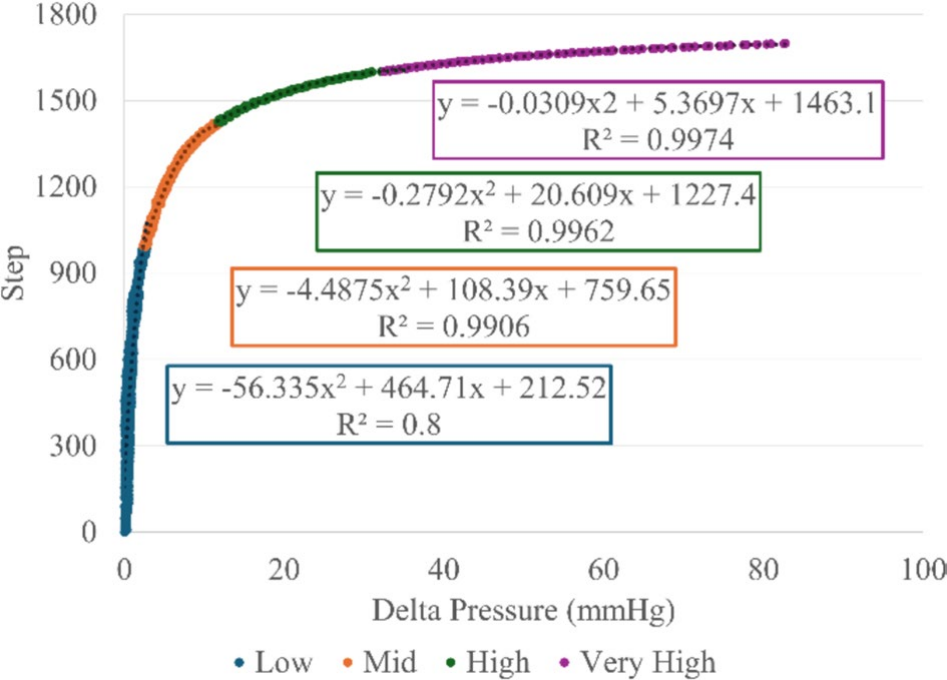
Vasopressor Control Module step vs delta pressure data plotted with quadratic regressions for each pressure region overlayed

Since the VCM is operated via stepper motor, this offers a convenient characterization of the VCM by defining the Delta Pressure in terms of motor step position. This was achieved using a piecewise set of quadratic regression functions shown in Fig. 5. A range of 1700 microsteps was found to cover a sufficient pressure window with a baseline position having no pressure effect and an observed maximum pressure delta of approximately 83 mmHg. Four pressure regions were defined including Low (0–3 mmHg), Mid (3–12 mmHg), High (12–35 mmHg), and Very High (35–83 mmHg). Each regression had R^2^ values of 0.99 or greater except for the Low region, which was 0.8. This was due to there being no significant change in pressure until ~ 300 steps at which point the more non-linear response began. A more refined baseline position can be defined in future iterations to potentially improve accuracy in this low delta pressure region, though the overall impact would likely be small.

**Fig. 6:**
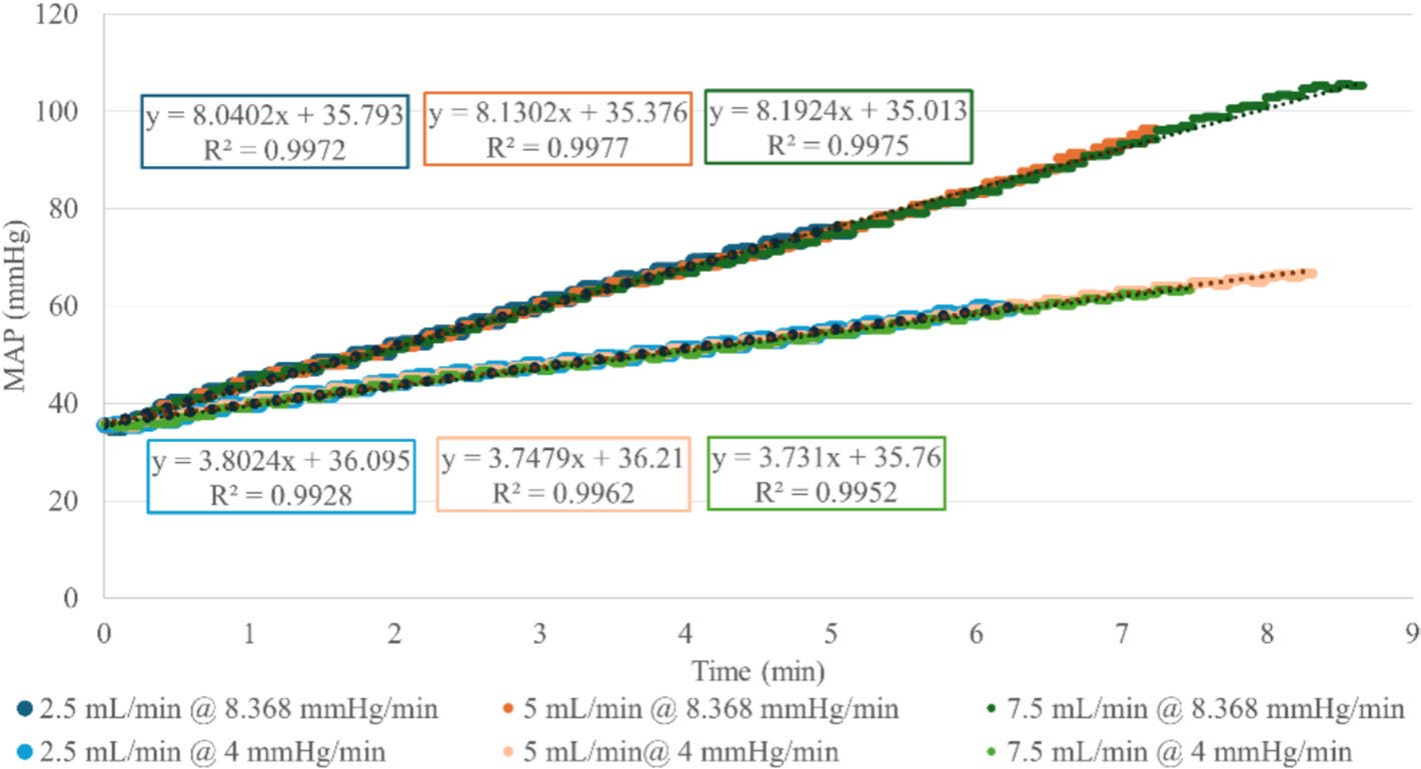
Pressure vs time data of the Vasopressor Control Module when administering select vasopressor infusion rate at two set pressure response rates with linear regression fits overlaid

These functions describing the step—delta pressure relationship provided a means to precisely control the Real Response of simulated vasopressor infusion rates but are insufficient for controlling the dynamic dMAP/dt behavior. However, solving each function for Delta Pressure and differentiating with respect to step position enabled the prediction of the step rate change of pressure based on the current step position (i.e., dP/dStep). Based on a desired dMAP/dt, the real-time result of dP/dStep was used to iteratively solve for the required motor step rate (i.e., dStep/dt). The system was confirmed across a range of vasopressor rates (2.5, 5, and 7 mL/min) at two dMAP/dt rates (8.368 and 4.0 mmHg/min). The time-based pressure results are shown in Fig. 6. Linear regressions of the pressure data had R^2^ values all above 0.99 demonstrating the system’s ability to generate a linear pressure response with respect to time, and the average pressure responses were accurate within 94% and 97% for 4.0 mmHg/min and 8.368 mmHg/min, respectively.

### Disturbance generator module

**Fig. 7:**
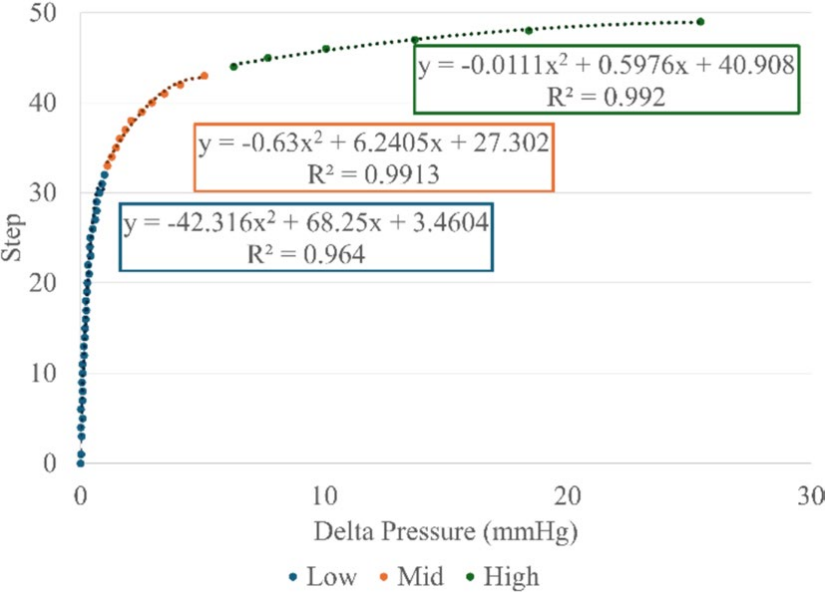
Disturbance Generator Module step vs pressure data plotted with quadratic regressions for each pressure region overlaid

The DGM was designed using a microcontroller, linear stepper motor, and a pinch valve. The stepper motor drove a lead screw which extended and contracted to actuate the valve, pinching the soft tubing of the fluid circulating portion of the HATRC loop to cause pressure perturbations (see Methods subsection “Modifications of the test platform” for more information). We characterized the DGM in a similar fashion to the VCM (Fig. 7), however the different motor and valve configuration had a lower resolution of both actuation and pressure. We used piecewise quadratic regressions to define the Step–Delta Pressure relationship across three pressure regions, Low (0–2 mmHg), Mid (2–11 mmHg), and High (11–60 mmHg). The total step range was limited to 45 microsteps and the maximum delta pressure observed was 60 mmHg. All the regressions had R^2^ values over 0.99. Using these functions, the module was given desired pressure deltas to perturb the system and actuated the motor to the necessary position.

### Controller proof-of-concept

**Fig. 8:**
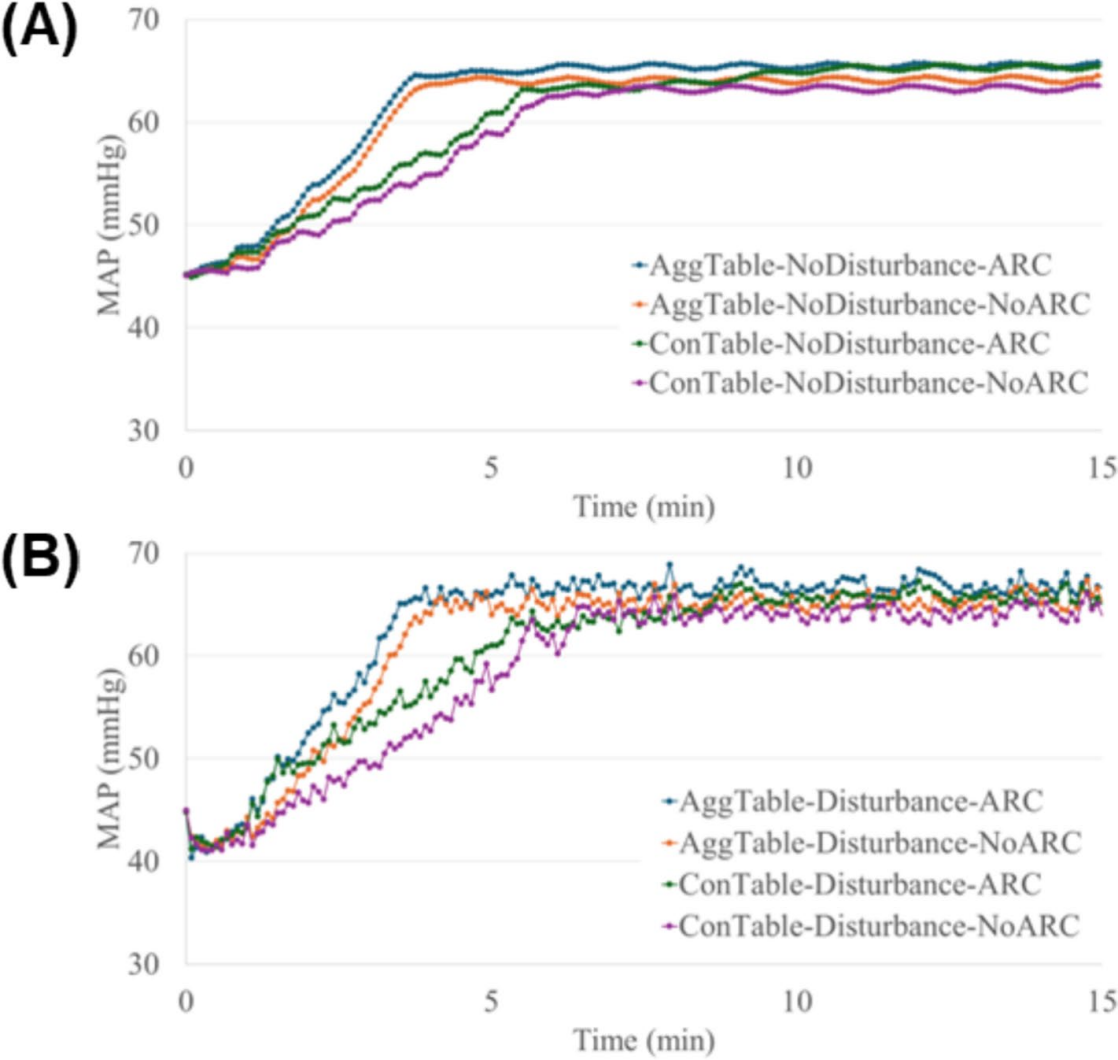
Average pressure vs time plots (*n* = 3) of proof-of-concept testing of an aggressive and conservative decision table controller with and without the aid of a secondary fluid infusion controller either **A** without pressure disturbance or **B** with pressure disturbance

The pressure vs time plots for the two controllers evaluated are shown in Fig. 8. Test runs were conducted with and without the inclusion of pressure disturbance as well as with and without the assistance of an adaptive resuscitation controller (ARC) running. Each scenario was run in triplicate, and the 3-run averages are shown in the figure. Overall, the effect of disturbance is visually evident, and the aggressive decision table (AggTable) had a visually faster response time compared to the more conservative decision table (ConTable) configuration (Fig. 8).

**Fig. 9:**
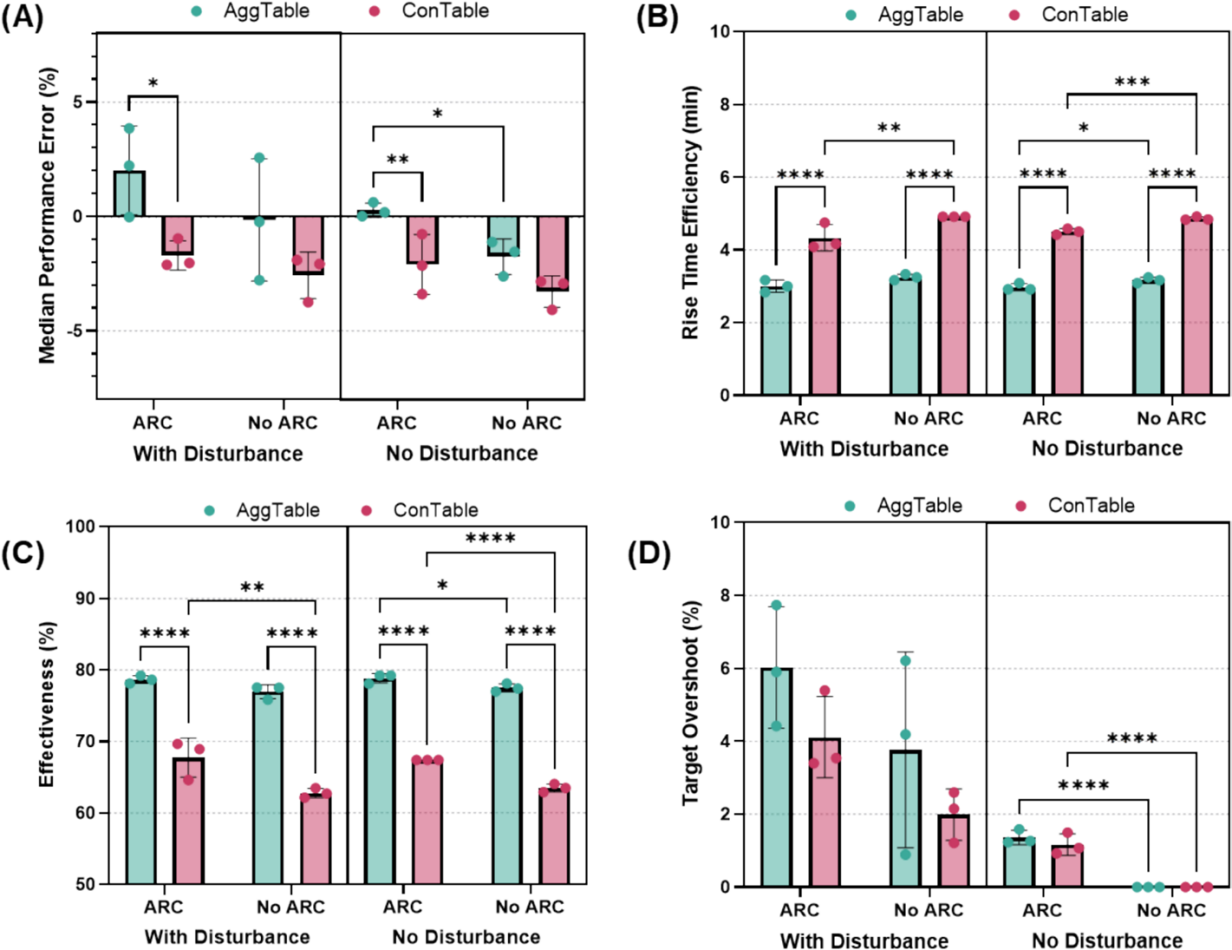
Average performance metrics (*n* = 3) for proof-of-concept vasopressor controller testing with the VCM module. Results are shown for **A** median performance error, **B** rise time efficiency, **C** effectiveness, and **D** target overshoot. Results for an aggressive and conservative decision table controller on with or without the aid of a secondary fluid infusion controller (ARC) and with or without disturbance are presented. Error bars denote standard deviation. Statistically significant differences between controller configurations for each metric are shown when present, as measured by two-way ANOVA, post hoc Fisher’s least significant difference test (* = *p* < 0.05; ** = *p* < 0.01, *** = *p* < 0.001, **** = *p* < 0.0001)

Four typical controller metrics were calculated to determine if statistically significant differences between specific controllers were identified (Fig. 9). Median performance error (MDPE, Fig. 9A) was most positive for AggTable, ARC-On, Disturbance-On at a value of 2.01% while the lowest MDPE was the ConTable, ARC-Off, Disturbance-Off at −3.28%. For rise time efficiency (Fig. 9B), the ConTable was consistently slower vs. Agg table (~ 4.6 min vs ~ 3.1 min, lower is better). The AggTable also performed better for the controller effectiveness metric (78% vs ~ 65%, higher is better, Fig. 9C). Lastly, we quantified a target overshoot metric (Fig. 9D) which tended to be larger for the AggTable (2.79% vs 1.82% ConTable), ARC-On (3.17% vs 1.44% ARC-Off), and Disturbance-On (3.97% vs 0.63% Disturbance-Off). The VCM module was capable of differentiating controller performance, highlighting its utility as a development platform for controller troubleshooting.

## Discussion

Hemorrhagic shock remains a leading cause of trauma-related death, which can often be prevented with restoration of blood pressure via fluid resuscitation. In some instances, resuscitation by fluid alone may not be possible due to fluid non-responsiveness or resource constraints limiting access to blood products. Vasopressor adjuvant therapies during fluid resuscitation may help in these instances if their rates can be titrated effectively. This can require careful monitoring of blood pressure, something that may not be practical in trauma care where MASCAL situations can limit personnel availability. As such, we are focused on developing closed-loop vasopressor administration controllers for trauma care, which require large datasets and extensive troubleshooting to be safe and effective. We previously developed closed-loop controllers for both whole blood and crystalloid infusion using a hardware-in-loop test platform for iterative testing and optimization prior to successful animal testing. Here, we explored the development of an adaptation to the hardware-in-loop system enabling its use for vasopressor controller development.

Analyses of previous animal data revealed a set of variables that could be used to characterize the physiological pressure response to a range of NE infusion rates. The VCM was then designed to mimic the characteristics of this response, simulating a patient receiving NE. The magnitude and rate of changes in MAP were of particular interest, and we confirmed the VCM’s ability to reproduce these features in a way that also allows customization by the user. We also implemented a module that mechanically added disturbance to the pressure signal to represent the effects of real-time data capture environments. Finally, we conducted proof-of-concept comparisons between two similar vasopressor controllers with and without pressure disturbances as well as with an assistive fluid resuscitation controller. Evaluating the controllers using standard metrics confirmed the platform’s usability for identifying potentially nuanced differences in performance between novel V-ARCs.

In addition to the waveform-specific metrics, we identified whether they were significantly impacted by inter-subject variability, drug infusion rate, as well as infusion rate direction. The pressure response rate, dMAP/dt, was the only variable found to be significantly affected by both the infusion rate and inter-subject variability, while the Real Response and Overshoot were only statistically different with respect to inter-subject variability. Lag Time did not demonstrate a significant effect from either inter-subject variability or infusion rate, but it was the only variable that showed a significant difference with infusion rate direction. This highlights the complexity of physiologic modeling as well as the need for varied and sometimes tailored testing.

The primary use for the VCM module is the systematic evaluation of closed-loop vasopressor administration and resuscitation. Proof-of-concept controller testing highlighted this utility by identifying feature and controller configuration differences with regard to controller performance. The more aggressive DT logic was significantly different from all conservative DT logics for rise time efficiency and controller effectiveness, highlighting how the aggressive logic reached target pressure more quickly and held at target for longer. Similarly, the pairing of V-ARC with ARC consistently led to target overshoot but had less effect on other performance metrics. These differences can be used to further tune controller logic to more effectively pair V-ARC and ARC together or to create less aggressive DT logic that reaches target pressure quickly while reducing overshoot effects. These results were extracted from a single scenario setup; with more scenario development to include different hemorrhage rates, varying starting pressures, as well as alternate target pressures, more controller performance trends can be extracted in a high-throughput manner prior to more costly, resource intensive animal testing.

Although the system was able to achieve its primary aim in distinguishing between vasopressor controllers, there is still room for improvement. The datasets used for the initial responsiveness characterization were relatively limited. More robust datasets would permit a wider range of patient conditions to be modeled and improve the accuracy of the predicted pressure response. For example, the current dataset does not provide information on how a severely hypotensive patient might respond to a high initial infusion rate of vasopressors or how the response might change using an alternative drug or with the presence of certain comorbidities. Thus, the need for animal testing is reduced, but not eliminated. The system was also developed around the infusion of a single concentration of NE where the dosage is regulated by infusion rate. A recharacterization can be conducted to generalize the NE dosing behavior to be compatible with alternative pumps, tubing sizes, and solution concentrations, however, in real-world use cases preparation of the equipment, such as priming the line and inputting certain specifications, will still be needed to ensure the administered dose assumed by the system is truly being delivered to the patient. There are also improvements that can be made to the VCM. The Overshoot variable was not modeled in this iteration of the module. As this was often a self-resolving feature that only lasted briefly, the more steady-state behavior is still reliable. However, the overshoot and median performance error metrics may score differently, particularly for controllers that utilize less frequent but larger increases in NE infusion rates. Some features like lag time and dMAP/dt were held constant for the proof-of-concept testing but can be improved by adding a factor of inter-subject randomness to the value(s) including logic that considers the rate- and direction-dependent deviations from the mean.

Another limitation with the VCM is the clinical context for which it was developed. The VCM is only as good as the data used to develop it, which was limited in scope to vasopressor therapy during hemorrhagic shock. Vasopressor responsiveness for other trauma states such as septic shock would not be applicable for the developed test platform. There are several studies reporting on the value in using metrics to predict circulatory dysfunction in septic shock, such as Ospina-Tascón et al. exploring the outcomes of patients with septic shock and their diastolic shock index [27] before starting vasopressors. The authors propose value in using a metric like the diastolic shock index to predict early need for vasopressors during septic shock. Such an implementation would require knowledge of vasopressor effects during septic shock; however, this is beyond the scope of the current VCM. In addition to vascular resistance, vasopressors also impact cardiac function and regional perfusion [28], neither of which are modeled by the simplified VCM. As the system was focused on only pressure responsiveness, other physiological changes such as diastolic arterial pressure, heart rate, or pulse pressure are not independently tunable for testing their responses through vasopressor use. As mentioned, with the appropriate reference data, the system could be modified to mimic these additional metrics, widening the system’s use case and improving the physiological realism of a multimodal response to vasopressor therapy.

## Conclusion

This work demonstrated the successful development and integration of a vasopressor control module into a hardware-in-loop resuscitation testbed. The platform was physiologically informed by animal datasets and proved to be effective at differentiating controller performance differences between two controller designs. In the broader context of trauma care, this work contributes to solving a pressing operational challenge—safe and effective vasopressor titration in resource-limited or MASCAL settings where continuous manual monitoring is impractical or impossible. The ability to rigorously test and refine autonomous control strategies in a controlled, high-fidelity environment supports the development of technologies that can extend critical care capabilities to austere and high-demand scenarios. Future work will incorporate additional hemodynamic parameters and explore multiagent resuscitation strategies, as well as develop a robust set of simulated trauma scenarios for down-selecting an assortment of V-ARC designs prior to animal testing. Ultimately, this approach can lead towards safer, faster, and more effective deployment of closed-loop vasopressor control in both military and civilian trauma systems.

## Methods

**Fig. 10:**
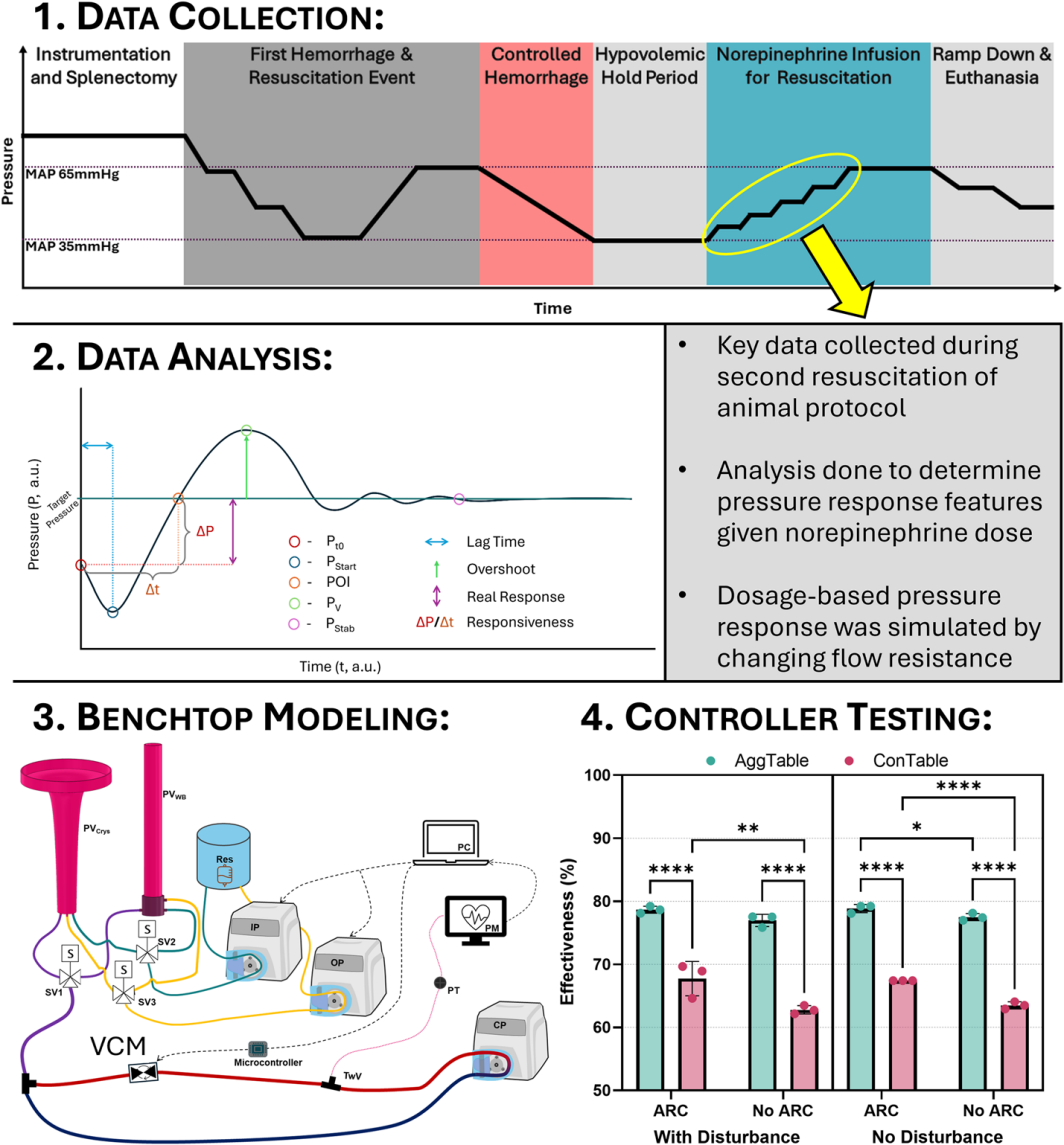
Methodological overview of the development of VCM and V-ARC testing. Shown is a timeline (Step 1) for the animal study from which norepinephrine datasets were used. Prior to vasopressor administration, a first hemorrhage and resuscitation event was performed of which data were not used in this analysis. The norepinephrine datasets were analyzed (Step 2) for key extracted features to characterize the dosage-based pressure response to a simulated flow resistance-based pressure response. The data analysis was used to develop and characterize (Step 3) the VCM module and integrate it into an existing benchtop hemorrhage resuscitation model. The developed platform was then used for initial proof-of-concept testing (Step 4) of V-ARC control schemes for automating vasopressor administration. Each aspect of the methods summarized in this figure are detailed in sub-sections

This section is structured as follows to describe the entire research study: description of animal data capture, preprocessing and data analysis, a short description of the current HATRC existing test platform, VCM development and integration into the HATRC platform for vasopressor functionality, and lastly, an overview of the proof-of-concept testing and the V-ARCs used. A diagram of key methodological sections is summarized in Fig. 10.

### Animal data capture and analysis

This research was conducted in compliance with the Animal Welfare Act, implementing Animal Welfare regulations and the principles of “The Guide for the Care and Use for Laboratory Animals” [29]. The protocol (A-24-003) received approval from the Institutional Animal Care and Use Committee (IACUC) at the United States Army Institute of Surgical Research where the research was conducted. The facility is fully accredited by AAALAC International. The experimental design utilized a refined swine model (*Sus scrofa domestica*) previously validated for similar applications [30]. Ten intact female Yorkshire crossbred swine, approximately four months old and weighing 40.6 ± 2.7 kg, were utilized. Swine were selected due to their well-documented physiological resemblance to humans, particularly in cardiovascular function [31, 32]. It should be noted that neither the VCM nor V-ARCs were used in animal experiments; animal data were used solely to inform the benchtop model, while all controller testing was conducted in the HATRC system.

Throughout the study, all subjects were maintained at a surgical plane of anesthesia and analgesia. Group membership for the captured data was assigned post hoc via drug exposure rather than a priori. As described previously [30], following initial instrumentation, subjects underwent a surgical splenectomy followed by a controlled stepwise hemorrhage (approximately 1–2 h) then resuscitation by the ARC using whole blood to recover the animals to a target MAP of 65 mmHg followed by holding at this pressure with ARC. After a total one-hour resuscitation period, a second controlled hemorrhage was performed to reach a hypovolemic MAP of 35 mmHg (approximately 30 min) guided by an automated algorithm termed “AutoBleed” [33]. Norepinephrine (NE), chosen based on the European trauma guidelines [34], was titrated to evaluate blood pressure responsiveness to vasopressor administration under hemorrhagic conditions. Concurrently, Lactated Ringer’s solution was infused at a fixed rate of 10 mL/min. NE (4 μg/mL) dosing followed a predefined escalation protocol (Table 1), with each rate held for 20 min or until the research team determined MAP had stabilized settled, i.e., ceased to increase, at that step, whichever happened sooner. The study events are summarized in Fig. 10.

**Table 1:** Stepwise norepinephrine infusion rates and corresponding dosages used for MAP management

Norepinephrine dose (mcg/min)	Infusion rate (mL/min)	Animal sample size increasing rate	Animal sample size decreasing rate
0.5	0.13	2	N/A
1	0.25	2	11
2	0.5	3	1
4	1	4	N/A
8	2	6	N/A
16	4	5	3
32	8	5	1
64	16	1	N/A

Sample sizes for each increasing and decreasing vasopressor rate are shown

Stepwise increases in vasopressor infusion continued until a sufficient response to vasopressors was reached, typically MAP reaching at least 65 mmHg. Next, the rate was reduced one step to assess weaning off effects. After MAP settled at the reduced step, experimental procedures were concluded. The subjects were humanely euthanized via intravenous administration of sodium pentobarbital (FatalPlus, Vortech; Dearborn, MI), in accordance with the American Veterinary Medical Association guidelines [35].

### Preprocessing and data analysis

Vasopressor infusion datasets from each animal were processed to look at the effect of vasopressor dosing on MAP. As such, the primary variables extracted for each swine were MAP and vasopressor infusion rate, each with respect to time (sampled at 1/5 Hz). A moving average was applied to the MAP signal to smooth the overall data trends during vasopressor administration. Data were sorted by vasopressor infusion rate steps, and within each of these groups, key fiducial points of interest were identified as follows and further described in Table 2: P_t0_, P_Start_, POI, P_V_, and P_Stab_.

**Table 2:** Summary of key fiducial markers from vasopressor datasets

Extracted feature	Description
P_t0_	Beginning of vasopressor delivery
P_Start_	The moment where the data crosses a specific slope threshold, indicating the first real positive (or negative, for down steps) response to the infusion rate
POI	The fifth consecutive data point wherein the absolute instantaneous slope is 50% below the local dMAP/dt maximum
P_V_	The vertex, maximum/minimum pressure achieved after P_Start_
P_Stab_	The stabilization point after PV, indicated by when the slope changes of 3 consecutive moving data windows are less than 0.05. If conditions are not met, the slope threshold is increased to 0.1

**Fig. 11:**
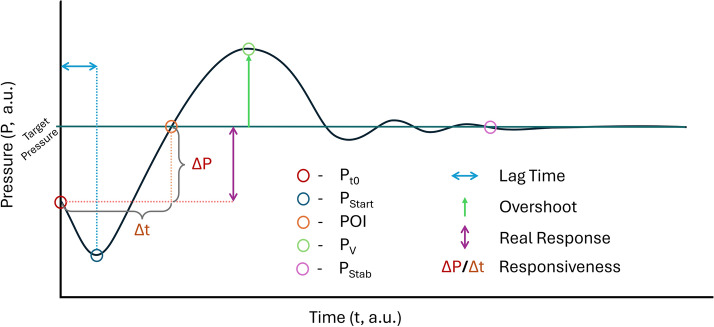
Representative diagram identifying key points used in vasopressor infusion data analysis. Key fiducial markers (P_t0_, P_Start_, POI, P_V_, and P_Stab_) and derived metrics (Lag Time, Overshoot, Real Response, and Responsiveness) are shown to illustrate what their measurements indicate for the dataset

Fiducial markers were used to calculate four additional derived metrics based on time and pressure differences between markers: Lag Time, Overshoot, Real Response, and Pressure–Time Responsiveness. Each of these is summarized in Table 3 and diagrammed in Fig. 11 to better visualize their meaning.

**Table 3:** Summary of derived metrics characterizing vasopressor responsiveness

Metric	Description
Lag time	An estimation of how long it took to see an actual pressure response from the time vasopressor was first administered at that rate. Calculated as time delta between P_t0_ and P_Start_
Overshoot	How high (or low) the pressure reaches relative to the level it ultimately settles at for any given infusion rate. Calculated as the pressure difference between P_V_ and P_Stab_
Real response	The total change in pressure from the initial baseline to its stable value. Calculated as the pressure difference between P_t0_ and P_Stab_
Pressure–time responsiveness (dMAP/dt)	The average slope between starting pressure and as the pressure approaches its vertex point. Calculated as the slope between P_t0_ and POI

Each of these markers and metrics were calculated for each vasopressor infusion rate change and summarized to quantify overall data trends related to vasopressor delivery during hemorrhagic shock resuscitation. In addition, combined plots of all vasopressor rates for each pig were generated, allowing for comparisons across different infusion rates within the same animal.

### Modifications of the HATRC platform

The HATRC system was previously designed for evaluating fluid resuscitation controllers. Our objective was to build a benchtop system capable of receiving fluid, analogous to whole blood and/or crystalloid solution, and responding with a change in a pressure signal that mimicked the fluid type-dependent responsiveness observed in a large animal hemorrhage study. We built a fluidic system with two primary “compartments”: a dynamic, circulatory loop and a hydrostatic, volume reservoir. The two compartments were then connected to permit volume changes in the reservoir to affect systemic pressure readings in the circulating loop. The reservoir was modeled by considering the height of the fluid column as directly proportional to the resulting hydrostatic pressure at its base, then deriving a function that relates the current volume to the height. To achieve this, we chose to use a circular cross-section whose diameter was a function of the height, which provided a convenient way to replicate both linear, i.e., where the diameter remains constant, and non-linear pressure–volume responses as desired [24, 25]. Computer-controlled pumps were then used to automate fluid resuscitation and simulated hemorrhage events by, respectively, adding and removing fluid from the reservoir. However, the system was incapable of simulating nonfluid volume-induced pressure changes.

In this work, we enabled vasopressor infusion functionality by incorporating a means to induce systemic pressure responses, independent of volume, that had similar features as those observed in swine treated with vasopressors. The vasopressor control and disturbance generator module development are described below, along with their communications integration with the greater HATRC test platform.

#### Vasopressor control module

To simulate the use of vasopressors in HATRC, we aimed to simulate a similar effect that vasopressors produce in the body, i.e., to raise pressure by increasing the flow resistance of the system. Unlike vasopressors, which achieve this effect primarily via systemic vasoconstriction, we implemented a device to variably choke the flow at a single point. The device comprised a stepper motor coupled with a needle valve which was integrated into the circulating portion of the loop. As the stepper motor tightened the valve, flow was constricted, resulting in increased pressure that was effectively independent of the fluid volume while still permitting volume-responsive pressure changes as needed.

Characterization of the device was conducted by actuating the stepper motor through a designated range of 1700 total steps in one-step increments. A one-second pause was held at each position for the first 1600 steps and was extended to 5 s for the final 100 steps. This allowed the pressure response to settle, at each position with the extended pause accommodating the greater pressure deltas per step that occurred as the flow became more occluded. The pressure vs step data were inverted then segmented into four regions which enabled a piecewise set of second-order polynomial regression functions to be defined as:

1 $$Step\left(\Delta P\right)=\left\{\begin{array}{c}f{\left(\Delta P\right)}_{1}, 0\le \Delta P<\Delta {P}_{1}\\ \dots , \dots \\ f{\left(\Delta P\right)}_{4}, \Delta {P}_{3}\le \Delta P<\Delta {P}_{max}\end{array},\right.$$

where $Step$ is the estimated stepper motor position required and $\Delta P$ is the desired pressure delta from baseline. The boundaries for each region were determined by optimizing the coefficients of determination of each regression function while minimizing the prediction error at the boundary points.

Since physiological pressure responses are not instantaneous, in addition to simulating the vasopressor-induced pressure changes, we also wanted to simulate the dynamics of the pressure over time by controlling the stepper motor actuation rate. Thus, the vertex forms of the quadratic $Step\left(\Delta P\right)$ functions were solved for pressure to obtain $\Delta P\left(Step\right)$ functions, and the first derivatives were solved for each to obtain $\frac{dP}{dStep}$ functions for each region. This provided a set of position-dependent functions which could then be used in the following equation:

2 $$\frac{dStep}{{dt}} = \frac{{\frac{dP}{{dt}}}}{{\frac{dP}{{dStep}}}},$$

where $\frac{dStep}{dt}$ is the necessary actuation rate of the stepper motor to produce a desired pressure response of $\frac{dP}{dt}$ based on inputting the current step position into the appropriate $\frac{dP}{dStep}$ function. With the system characterized as such, we have precise control over pressure dynamics that are relatively independent of volume and that can be theoretically tuned for any vasopressor. However, the system is currently tuned to only replicate the response features when NE was used; further data collection would be needed to model alternative vasoactive drugs. In addition to this, the overall pressure dynamics are very stable, lacking the noise and variability typically seen in physiological data. For this reason, we incorporated a module to introduce pressure perturbations having a similar distribution to that observed in swine.

#### Disturbance generator module

To generate disturbances, a stepper motor-driven linear actuator was employed to introduce ± 7 mmHg pressure fluctuations by pinching the flow-loop tubing. The actuator was first positioned at a median “home” step index, then driven above or below this point to manipulate the pressure. The disturbance profile was obtained by an analysis of the MAP signal from three subjects. Between 2–4 sample segments were extracted from each subject and the standard deviations were found. Sample segments were taken from maintenance periods of the protocol when the MAP was relatively stable and there were no active hemorrhage or resuscitation events occurring. To simulate the most challenging disturbance conditions, the largest standard deviation of ~ 7 mmHg was used to characterize the disturbance distribution.

To determine the home position and relate the step indices to pressure, the actuator was cycled through a range of 45 steps in single-step increments. Each step was held for five seconds for the first 30 steps, and 15 s for the final 15 steps. The sequence was then mirrored in reverse order. During this sweep, steady-state pressure was recorded at each step. The resulting pressure vs step relationship was subdivided into three regions, each modeled by a second-order polynomial, similar to the VCM. These piecewise regressions received a desired delta pressure (ΔP) as an input and output the corresponding step index required to achieve that ΔP from the baseline. The home position step was defined as corresponding to a ΔP of ~ 9 mmHg from the absolute baseline, allowing both positive and negative pressure disturbances to be effected. With the characterization complete and the home position defined, a generated sequence of random ΔPs was passed through the piecewise polynomial functions to compute the target step. The actuator was then driven to the target step, held for one second, then another ΔP value was passed. This method produces controlled, randomized perturbations into the system.

#### HATRC platform and communications

The primary HATRC system runs on a PC in MATLAB (MathWorks, Natick, MA, USA). The VCM and DGM’s microcontrollers were integrated into HATRC using specialized libraries and serial communication protocols. The system pressure is collected using a data acquisition system (PowerLab, ADinstruments, Sydney, Australia) and sent to the HATRC system which provides this as an input to the V-ARC control script. The V-ARC in use determines the vasopressor infusion rate and sends this as an output to the HATRC system. The desired infusion rate is then transmitted via serial communications to the VCM’s microcontroller where it is first converted to a predicted delta pressure and the stepper motor is sent to the corresponding position as determined by the characterization functions. All modules operate with a 5-s sampling period for compatibility with the HATRC system.

### Vasopressor controller proof-of-concept testing

To better understand the testing procedure, each controller logic is first introduced, followed by how proof-of-concept testing was configured for the VCM module.

#### ARC overview

For automated fluid administration, we utilized a previously developed adaptive resuscitation controller, or ARC which has been successfully verified in a large animal model of hemorrhagic shock [30]. Briefly, ARC is driven by MAP as its primary input variable with a target MAP value set as the goal for directing fluid therapy. An initial bolus of 100 mL of fluid is administered over one min to estimate pressure-fluid responsiveness which is fitted with a linear regression model. With that responsiveness, ARC calculates a flow rate to allow for an increase in pressure at a rate of 2 mmHg/min for the assistive model used here. Fluid responsiveness is continuously re-calculated during resuscitation allowing for adjustment of fluid rates to maintain the pressure response rate. Flow rates are halted to 0 mL/min once target MAP is reached.

#### Decision table logic overview

The Decision Table V-ARC (DT V-ARC) used a predefined set of vasopressor infusion rates. An error signal, obtained by taking the difference between the current and target MAP, was used to determine whether to increase vasopressor rate. A 30-s sampling time was used, and the infusion rate was increased by one step on the table if a positive error was received. Once the target pressure was reached or exceeded, the system held the current rate. The initial version of the controller was modified by adding half-steps to each dosage change thus having a more conservative ramp rate compared to the initial version which had a more aggressive ramp rate, and the two versions were named accordingly.

#### Scenario development

To assess the performance of these preliminary V-ARC controllers and how the system parameters may impact the performance, a set of four simple test scenarios were derived. The HATRC system was initialized at a starting MAP of 45 mmHg, and a target MAP of 65 mmHg was used for each 15-min run. Tests were conducted in triplicate for all permutations of with or without disturbance and either utilizing or not utilizing a secondary ARC alongside the V-ARC controller. Conventional performance metrics such as median performance error (MDPE), target overshoot, controller effectiveness, and rise time efficiency were calculated to characterize performance differences [26].

### Statistical methods

For the analyzed swine data, we evaluated if there were significant differences between each derived metric and their impact on vasopressor activity. Specifically, this was split into four separate analyses looking at whether vasopressor concentration and pig variability significantly impact lag time to pressure increase, pressure increase or real response, overshoot, and pressure response rate (Fig. 10). Each of these were evaluated by two-way ANOVA with data separated by vasopressor infusion rate and swine subject. They were analyzed separately for differences between increase and decrease NE infusion rate steps on each of the 4 metrics, using Welch’s t-test for these analyses. All data sets were identified as normally distributed using Shapiro–Wilk tests. Statistically significant effects were determined as p-values less than 0.05 and are indicated in the text and figures when applicable.

For the proof-of-concept controller testing, two-way ANOVA, post hoc Fisher’s least significant difference test was used to assess difference between controller configurations for each performance metrics. The two factors for this analysis were DT tuning (aggressive vs. conservative) and ARC state (on or off). Each disturbance configuration and performance metric were analyzed separately. Statistically significant effects were determined as p-values less than 0.05 and are indicated in the text and figures when applicable.

## Data Availability

The data presented in this study are not publicly available because they have been collected and maintained in a government-controlled database located at the U.S. Army Institute of Surgical Research. This data can be made available through the development of a Cooperative Research and Development Agreement (CRADA) with the corresponding author.

## Declarations

### DoD disclaimer

The views expressed in this article are those of the authors and do not reflect the official policy or position of the U.S. Army Medical Department, Department of the Army, DoD, or the U.S. Government.
